# The Influence of EEG References on the Analysis of Spatio-Temporal Interrelation Patterns

**DOI:** 10.3389/fnins.2019.00941

**Published:** 2019-09-12

**Authors:** Wady A. Ríos-Herrera, Paola V. Olguín-Rodríguez, J. Daniel Arzate-Mena, Maria Corsi-Cabrera, Joaquín Escalona, Arlex Marín-García, Julieta Ramos-Loyo, Ana Leonor Rivera, Daniel Rivera-López, José F. Zapata-Berruecos, Markus F. Müller

**Affiliations:** ^1^Centro de Ciencias de la Complejidad, Universidad Nacional Autonoma de México, Mexico City, Mexico; ^2^Instituto de Ciencias Nucleares, Universidad Nacional Autonoma de México, Mexico City, Mexico; ^3^Instituto de Investigación en Ciencias Básicas y Aplicadas, Universidad Autónoma del Estado de Morelos, Cuernavaca, Mexico; ^4^Research Unit in Neurodevelopment, Institute of Neurobiology, National Autonomous University of Mexico, Querrétato, Mexico; ^5^Centro de Investigación en Ciencias, Universidad Autónoma del Estado de Morelos, Cuernavaca, Mexico; ^6^Instituto de Neurociencias, Universidad de Guadalajara, Guadalajara, Mexico; ^7^Instituto Neurológico de Colombia, Medellin, Colombia; ^8^Centro Internacional de Ciencias A. C., Universidad Nacional Autonoma de México, Cuernavaca, Mexico

**Keywords:** electroencephalography, EEG reference, multivariate analysis, functional network, time series analysis

## Abstract

The characterization of the functional network of the brain dynamics has become a prominent tool to illuminate novel aspects of brain functioning. Due to its excellent time resolution, such research is oftentimes based on electroencephalographic recordings (EEG). However, a particular EEG-reference might cause crucial distortions of the spatiotemporal interrelation pattern and may induce spurious correlations as well as diminish genuine interrelations originally present in the dataset. Here we investigate in which manner correlation patterns are affected by a chosen EEG reference. To this end we evaluate the influence of 7 popular reference schemes on artificial recordings derived from well controlled numerical test frameworks. In this respect we are not only interested in the deformation of spatial interrelations, but we test additionally in which way the time evolution of the functional network, estimated via some bi-variate interrelation measures, gets distorted. It turns out that the median reference as well as the global average show the best performance in most situations considered in the present study. However, if a collective brain dynamics is present, where most of the signals get correlated, these schemes may also cause crucial deformations of the functional network, such that the parallel use of different reference schemes seems advisable.

## 1. Introduction

Although electroencephalography (EEG) is one of the most popular techniques for the study of brain dynamics, the search for the ideal reference point still remains. For principal reasons it is impossible to measure the magnitude of the electrical potential at a given location in space, so that only potential differences between two positions are experimentally accessible (Jackson, 1999). Given that dynamical changes of the electrical brain activity cause only small changes of the potential at the scalp surface, which are of the order of microvolts, it is highly desirable that the electrical potential at the reference point stays precisely at a constant value in order to avoid erroneous alternation of the time dependency of the signal at the recording side. Given that the fluctuations of the electrical potential of external fields are in general larger or at least of the same order of magnitude as those generated by the electrical activity upon the scalp, one pragmatically seeks for an optimal solution within the system, although it is well known that the human body does not contain an electrically neutral location (Nunez and Srinivasan, 2006). Therefore, the impossibility to encounter an ideal solution remains clear beforehand.

The first studies about the issue were reported more than half a century ago (Goldman, 1950; Offner, 1950), and since then a multitude of important contributions have been published, each with a different angle of view on this problem (see e.g., Osselton, 1965; Hjorth, 1975; French and Beaumont, 1984; Bertrand et al., 1985; Fein et al., 1988; Rappelsberger, 1989; Lemos and Fisch, 1991; Travis, 1994; Nunez et al., 1997, 1999; Zaveri et al., 2000, 2006; Rummel et al., 2007; Hu et al., 2010, 2018; Kayser and Tenke, 2010, 2015; Nunez, 2010; Chella et al., 2016; Lei and Liao, 2017; She et al., 2017; Trujillo et al., 2017). Motivated by the fact that the study of the functional network, constructed by some preferential bivariate measure applied to brain signals, gained a lot of attention during the last decades, we focus here mainly on the effect that a particular reference choice may have on the topological properties as well as the time evolution of the interrelation pattern between different brain signals.

A large body of evidence has been accumulated showing that estimates of bivariate measures may be crucially affected by the chosen reference scheme, such that numerical results become “virtually uninterpretable” (French and Beaumont, 1984). For instance, coherence values estimated from EEG signals with linked earlobes are up two time larger as those when the Laplacian montage has been used (Travis, 1994). By employing autoregressive models to simulate EEG signals with well defined power spectra and specific mutual interrelations, it turns out that coherence values estimated under the influence of the global average are most useful when signals are independent or nearly independent (Rappelsberger, 1989).

It is a matter of fact that the integral over the surface of a volume conductor of the electrical potential is precisely zero (Bertrand et al., 1985; Jackson, 1999). Therefore, if a sufficiently high number of electrodes homogeneously placed around the whole brain are used, the global average should provide a nearly constant reference signal. However, if signals are mutually independent, also with the partial and sparse covering of the 19 electrode international 10/20-system one should expect approximately an equivalent quality. If on the other hand active electrodes are correlated, or, if the power of a few active electrodes is notably larger than that of the remaining ones, the global average is no more electrically inactive and may cause disturbances (Fein et al., 1988). Therefore, the averaged earlobe signals are recommended in more general terms (Rappelsberger, 1989).

Some authors (Nunez et al., 1999) conclude that close bipolar as well as the Laplacian reference eliminate undesired volume conduction effects but systematically underestimate coherence by the spatial filtering effect. Also in Zaveri et al. (2006) it is advised to use the bipolar montage with caution in time series analysis. Instead, in Nunez et al. (1999) the authors find that for large-scale interrelations, the global average as well as linked mastoids provide reasonable “semi-quantitative” coherence estimates, while the usage of an active electrode, e.g., Cz, as a reference point is discarded (Nunez et al., 1997). A similar conclusion is drawn in Zaveri et al. (2000). Here also the earlobe reference is considered to have a limited impact on coherence measurements if the power of the reference signal is considerably smaller than that of active electrodes, a supposition that is not necessarily true for extracranial recordings.

In Rummel et al. (2007) this issue was discussed in the context of Random Matrix Theory. Using multivariate techniques stemming from this research field (Laloux et al., 1999; Plerou et al., 1999, 2002; Müller et al., 2006) they tested the influence of 6 popular reference schemes on the eigenvalue spectra of the zero-lag cross-correlation matrix. Furthermore, a correction algorithm is derived, which is proposed to minimize such perturbations to a large extent. As a result it turned out that the distortions caused by the global average were less pronounced than those of other schemes, and the proposed correction algorithm worked almost perfectly in this case.

For the case of multivariate analysis of EEG data, which has gained increasing interest during the last decades, in Chella et al. (2016) the influence of EEG references on the connectivity and graph theoretical measures has been probed by using the imaginary part of the coherency. The authors conclude that significant differences arise by using different reference schemes for the estimation of scalp EEG functional connectivity and, consequently, a significant influence on the estimation of the topological properties of the functional network, as assessed by graph theoretical metrics, has been detected. The authors advise that the comparison of results derived in different laboratories using different data acquisition and pre-processing techniques should be undertaken with caution.

In the present study the authors present and quantify the spatiotemporal distortions caused by different EEG-references, using linear cross-correlations, as well as phase synchronization as a representative for a linear and a non-linear bivariate measure, respectively. To this end, we employ artificial data derived from theoretical models, where the interrelation pattern between data channels can be perfectly controlled. In order to investigate time dependencies we use additionally three types of real EEG-recordings: Sleep EEG from healthy subjects, a peri-ictal recording of an epileptic focal onset seizure and recordings of clinically healthy subjects with open and closed eyes while hearing music. We chose these examples because they show striking differences in terms of the morphology and the interrelation pattern of the signals. Furthermore, the peri-ictal transition of an epileptic seizure as well as the transitions between different sleep stages are highly non-stationary, and thus they allow probing for the time dependency of the functional network derived for different reference schemes. Specifically, these recordings are used to derive realistic EEG-references, which can then be applied to the model data in order to visualize their effect on the correlation pattern and the power spectra in a more objective way.

For this purpose, we probed for those reference schemes already considered in Rummel et al. (2007) and included additionally the median reference as a non-parametric version of the global average. In principle, one should expect that the influence of the global average and the median is of comparable size. However, the non-parametric character of the median ensures a higher robustness against outliers, since the global average can be quite sensitive to muscle artifacts that are restricted to only a few signals, but enter with high amplitude, while the median remains unaffected from such outliers.

In Yao (2001), a new reference montage has been proposed which is superior than other schemes in several aspects (Qin et al., 2010; Liu et al., 2015; Lei and Liao, 2017; She et al., 2017; Trujillo et al., 2017; Hu et al., 2018). However, the so called Reference Electrode Standardization Technique (REST) requires a precise head model in order to simulate electrically active sources. Such estimates improve in general with an increasing number of electrodes and may show essential deficiencies when only 64 or less electrodes are used (Acar and Makeig, 2013). Even when the location of sources can be almost precisely determined, the lack of knowledge of resistivity and the anisotropy of brain and skull tissues constitute major error sources (Nunez, 2010). Additionally, these parameters might differ across subjects. Furthermore, source distributions might change drastically during recording time. For instance, dominant sources alter drastically during the transition from wake state to sleep (Steriade, 2003) and during sleep cycles (Pace-Schott and Hobson, 2002; Steriade, 2003; Dang-Vu et al., 2010; Brown et al., 2012; Jones, 2019). Even the much longer circadian rhythm (Dijk and Czeisler, 1995; Pace-Schott and Hobson, 2002; Brown et al., 2012) is regulated by specific neuronal circuits. The same is true for the peri-ictal transition of epileptic seizures. While during its initial stage the seizure onset zone may play a major role in the generation of particular brain rhythms, the dynamical evolution of seizures (Geier et al., 2015) and, in particular, the collective termination of focal onset seizures seem to be orchestrated by widely distributed well-coordinated network activity (Schindler et al., 2006, 2008). Consequently, the source distributions are drastically altered during epileptic events (Alarcon et al., 1994). In the cases of such highly nonstationary recordings, it seems advisable to properly design a head model individually for different epochs. For these reasons, in the present study we solely focus on model-free reference schemes, which seems to be of more practical usage when only 19 electrodes of the 10/20 systems are considered for the measurement of highly nonstationary events.

The paper is organized as follows. In the next chapter, the method section, we describe the reference schemes considered in this work, the numerical test frameworks and we describe the EEG data and the interrelation measures used. Then, in section 3, we present the numerical results concerning the spatial deformations of several well defined correlation patterns and check for possible consequences of re-referencing. It is supposed that earlobe signals are not sensitive for neural activity and are thus independent from those of the active electrodes. Also in our model, calculation-simulated earlobe signals are derived independently. However, we probe for the validity of this assumption in a separate section. Finally, several applications on real world data are discussed. Namely, we study the peri-ictal correlation dynamics of a focal onset seizure, we consider deep sleep as well as paradoxical sleep and check for vulnerability to muscle artifacts. In a final section, we draw our conclusions.

## 2. Methods

### 2.1. Reference Schemes Considered in the Present Study

The time–resolved values of an electroencephalographic recording (*Y*_*i*_(*t*)) represent the difference between the electrical potential measured at the position of an scalp electrode (*X*_*i*_(*t*)) and some reference potential *R*_*i*_(*t*) measured elsewhere, viz.:

(1) $$\begin{array}{r} Y_{i}\left(t\right)=X_{i}\left(t\right)-R_{i}\left(t\right) \end{array}$$

where *i* = 1, …, *M* denotes the index of the active electrodes. In order to define a reference point *R*_*i*_(*t*) different schemes have been established. Here we consider the most common ones:

1. Average of two active electrodes (*F*3*F*4): The average of the two scalp electrodes (*F*3 and *F*4) are taken as reference for all others:
  (2) $$\begin{array}{r} R\equiv R^{F}=\left(X_{F3}+X_{F4}\right)/2. \end{array}$$
  Sometimes only one scalp electrode like *Cz* is used instead, but pitfalls disclosed for the *F*3*F*4 reference are similar to those when only one active electrode is chosen.
2. Earlobes average (*A*1*A*2): The average of the two earlobe electrodes *A*1 and *A*2 is taken as reference:
  (3) $$\begin{array}{r} R\equiv R^{A}=\left(X_{A1}+X_{A2}\right)/2. \end{array}$$
  It is supposed that this reference signal is not substantially influenced by the brain activity and thus it is (almost) independent from scalp electrodes. Our numerical simulations are also based on this assumption, where earlobe recordings are derived as independent signals. Furthermore, in the numerical test framework used by us to simulate active electrodes as well as reference signals, this scheme is equivalent to the mastoid reference. Occasionally, only one earlobe signal is used.
3. Contra-lateral reference (CL): The earlobe electrodes *A*1 and *A*2 are taken separately as reference for the right and left hemisphere, respectively.
4. Hjorth montage (H): For a given electrode the weighted average of neighboring electrodes is taken as a reference. The weights depend on the relative distance between the electrodes, for details see Hjorth (1975) and Gordon and Rzempoluck (2004).
5. The global average (gav): The Reference signal is the average over all *M* scalp electrodes:
  (4) $$\begin{array}{r} R\equiv R^{G}=\frac{1}{M}\overset{M}{\underset{i=1}{\sum }}X_{i}. \end{array}$$
  This scheme is motivated by general principles (Bertrand et al., 1985; Jackson, 1999) and further by the assumption that dynamical aspects of the brain activity are averaged out such that one ends up with an (almost) constant reference signal.
6. Median (M): The median reference is the non-parametric version of the global average scheme. At each time step the median *R*^*M*^ of all EEG-channels (*M*) is taken as a reference. Hence, for the scheme of 19 electrodes, only one scalp electrode is used as a reference point, but it may change at any instant (Müller et al., 2011, 2014; Olguín-Rodríguez et al., 2018).
  (5) $$\begin{array}{r} R\equiv R^{M} \end{array}$$
7. Bipolar montage (bp): Finally, bipolar montages have ample clinical applications, such as for diagnostic purposes of, e.g., epilepsy. In the present study we use the following scheme: we close the left (*Fp*1 − *F*7, *F*7 − *T*3, *T*3 − *T*5, *T*5 − *O*1, *Fp*1 − *F*3, *F*3 − *C*3, *C*3 − *P*3, *P*3 − *O*1) and right hemisphere (*Fp*2 − *F*8, *F*8 − *T*4, *T*4 − *T*6, *T*6 − *O*2, *Fp*2 − *F*4, *F*4 − *C*4, *C*4 − *P*4, *P*4 − *O*2) of the standard surface EEG to rings and couple the three central electrodes: *Fz* − *Cz* and *Cz* − *Pz*. This choice leads to a total of *M*′ = 18 data channels.
  (6) $$\begin{array}{r} R\equiv R^{B}. \end{array}$$

### 2.2. Numerical Test Frameworks

In order to put this study on solid statistical footings, we synthesized multivariate data sets from theoretical test models, where strength as well as the spatial distribution of the cross-correlations can be perfectly controlled. Then, deviations from such well defined correlation structures, provoked by some reference signal, can be precisely evaluated. Here we chose multivariant *N*_*f*_-tori (Müller et al., 2005) as a test framework, because this model bears the additional advantage that one may adjust independently the amount of cross-correlations between data channels as well as the power spectrum of each signal.

*N*_*f*_-tori are sums of *N*_*f*_ harmonic oscillations with incommensurate frequencies, and as such they can be understood as a kind of discrete Fourier decomposition of a whatever time series:

(7) $$\begin{array}{r} X_{k}\left(t\right)=\overset{N_{f}}{\underset{l=1}{\sum }}A_{kl}\sin\left(2\pi f_{l}t+\delta_{kl}\right), \end{array}$$

with *k* = 1, …, *M* data channels, which mimic the electrode signals. Given the close relationship of the *N*_*f*_-tori to the Fourier theory, it puts this test framework on very general grounds. Via the Wiener-Khinchin theorem, it remains clear that all linear univariate properties are fixed via the selection of the amplitudes *A*_*kl*_, while non-linear autocorrelations and simultaneously linear as well as non-linear cross-correlations are governed by the initial phases δ_*kl*_. However, for a given realistic situation, the distribution of the δ_*kl*_ might be quite complicated. Here we used a simple but transparent strategy in order to generate different interrelation patterns.

Hence, the *A*_*kl*_ are the amplitudes of the harmonic oscillations, while the δ_*kl*_ are the phases at *t* = 0. Note, in Equation (7) the *f*_*l*_ take the same values for all *M* signals, while amplitudes *A*_*kl*_ and initial phases δ_*kl*_ are chosen individually for each data channel. The amplitudes *A*_*kl*_ fix the power spectra and therefore the amount of random correlations within the multivariate dataset (Müller et al., 2005). The initial phases δ_*kl*_, on the other hand, permit the control of genuine correlations between pairs or groups of signals (Müller et al., 2005). If the δ_*kl*_ are uniformly distributed between zero and 2π the *X*_*k*_(*t*) are completely uncorrelated, because each phase relationship between data channels occurs with equal probability, and frequency ranges with specific phase relations are absent. Any deviation from this uniform distribution implies phase relationships between time series and gives rise to the occurrence of genuine correlations (Müller et al., 2005). In the present study, correlations are generated via Gaussian distributions modulo 2π with mean μ_δ_ = π and standard deviation σ_δ_ ∈ [0, 2π] such that on the average a constant phase difference is produced (Hramov et al., 2005).

For each signal of the multivariate set, *N*_*f*_ = 5000 frequencies are randomly selected using a uniform distribution between 0.1 and 100 Hz. Here a time unit is arbitrarily chosen as 256 sampling points, and the amplitudes are fixed according to a Gaussian distribution:

(8) $$\begin{array}{r} A_{kl}\left(f_{l}\right)=\frac{1}{\sqrt{2\pi }\;\sigma_{A}}\exp\left[-\frac{1}{2}{\left(\frac{f_{l}-\mu_{A}}{\sigma_{A}}\right)}^{2}\right], \end{array}$$

where μ_*A*_ and σ_*A*_ are, respectively, the center and the standard deviation of the Gaussian. With that choice we simulate qualitatively band-pass filtered EEG data. Note, band-pass filtering is mandatory if one aims to apply phase-synchronization measures, which require mono-component narrow-band signals (Picinbono, 1997; Chavez et al., 2006; Rios Herrera et al., 2016) To each of the data channels of the *N*_*f*_-tori we assigned an electrode of the 10/20-system in order to define the Hjorth and bi-polar reference adequately. By means of the δ_*kl*_ we generated 8 different correlation structures, each set consisting of 21 signals, where 19 simulate the active scalp electrodes and the two remaining ones mimic the earlobe electrodes (*A*1, *A*2), or the mastoid signals, respectively. A detailed description of the 8 models is given in Table 1.

**Table 1:** Correlation patterns generated by *N*_*f*_-tori.

**Model**	**Standard deviation (σ_δ_)**	**Correlated channels**
1	–	None
2	π/5	All signals
3	π/20	1–9
4	π/20	1–10
5	π/20	3 and 4
6	π/20	3, 5, 7, 9, 11, 13, and 15
7	π/20	Two clusters (1–8) and (9–16)
		with mean phase difference of π/2
8	π/20	Three weakly interrelated correlation cluster
		(1, 2), (8, 10), and (18, 19) centered at
		π/20, 11π/40 and 9π/40, respectively

Model 1 and 2 simulate the limit cases where none or all data channels are correlated, respectively. In models 3−6 correlations are induced within a subset of the 19 signals (one single cluster). Two mutually uncorrelated clusters are generated in model 7. For this purpose, the centers of the Gaussian distributions of the initial phases of the two clusters are displaced by π/2. Finally, in model 8 three small correlation clusters are formed. The centers of the Gaussian probability distributions used to select the initial phases are chosen as π/20, 11π/40, and 9π/40. Hence, the three correlation clusters are mutually correlated.

### 2.3. Description of the EEG-Data

We analyze three examples of electroencephalographic recordings. The first signal was acquired at the Sleep Laboratory of the Faculty of Psychology of the Universidad Nacional Autónoma de México. It was taken from a 26-year-old, clinically healthy male subject during the whole night after giving written informed consent. The recording considered here was obtained during the second night in the laboratory in order to avoid irregularities of the first night sleep in a new environment. Prior to the study, the subject had a structured clinical interview and kept a 15-day sleep log, confirming a regular sleeping habit without any symptoms of sleep disorders. The experiment was approved by the Ethical Committee of the Faculty of Medicine of the Universidad Nacional Autónoma de México following the ethical standards of the Declaration of Helsinki (1964). Standard polysomnography (PSG) and a standard scalp EEG were recorded at Fp1, Fp2, F3, F4, F7, F8, C3, C4, T3, T4, T5, T6, P3, P4, O1, O2, Fz, Cz, and Pz of the 10/20 International System (Lesser, 1986) referenced to A2 with a Grass 8 - 20 polygraph with filters set at 0.1 and 70 Hz for EEG, at 10 and 70 for EMG, and 0.3 and 70 Hz for EOG. Additionally, earlobe A1 referenced to A2 was also recorded. Beside the construction of the functional network spanned by all active electrodes, this scheme allows additionally the estimation of possible correlations between them and the earlobe signal A1. Hence, one is able to probe the independence of earlobe signals.

All-night PSG data were digitized and stored with a 1,024 Hz sampling rate using a 12-bit A–D converter of the GRASS-GAMMA acquisition program. Wakefulness and sleep stages were identified by standard procedures using 30-sec epochs (Rechtschaffen and Kales, 1968) by an experienced specialist in sleep recordings (MC-C). For the data analysis we applied a low pass filter (4th order Butterworth) suppressing all frequencies above 25 Hz and downsampled the recording to 128 Hz effective recording frequency. Then a band-pass for the delta-band (0.5-3.5 Hz), the fast beta-band (18–25 Hz) and the broad band (1–40 Hz) was applied. Thereafter, the signals were reference transformed.

In particular, we analyzed sleep stage 4 and Rapid Eye Movement (REM) sleep because of the strikingly different morphology and functional connectivity of these sleep stages. Sleep stage 4 resembles a more collective state dominated by large amplitude slow waves, while recordings during REM sleep have a larger contribution of fast frequency components and are in general more similar to the awake state. In a first example we considered in total 50 epochs from this data set, each with a 30-s duration, which are consecutive during the first sleep cycle in the case of sleep stage 4 and were taken from the last two sleep cycles in the case of REM-sleep. In a second calculation we considered the whole night recording with a 30-s running window.

The second example is a 5-min recording of a 30-year-old female epilepsy patient, measured in the Instituto Neurologico de Antioquia in Colombia in accordance with its surgery protocol. The EEG contains the peri-ictal transition of a secondary generalized focal onset seizure with a tonic-clonic seizure offset and a duration of 70 s. The recording was obtained by using a Cadwell Inc with 32 channels with the settings of the international 10/20 system. However, only the standard 19 channels were used, referenced to A1, with a recording frequency of 250 Hz, filtered between 0.1 to 70 Hz and a Notch filter at 60 Hz. The acquisition program is called Easy III. Ten-second epochs have been evaluated from an experienced neurophysiologist (JFZB). The patient signed a written consent and the protocol was approved by the Ethics Committee of the Clinical Research Neurological Institute of Colombia. Before we analyzed the data set with a 10-s, non-overlapping window, the data were band-pass filtered between 0.5 and 25 Hz by using a 4th-order Butterworth filter, employed in forward and backward directions in order to avoid artificial phase shifts.

The third type of EEG data used in this paper were recorded from healthy subjects (four women and eight men; age range 23–38 years) in the Laboratory of the Psychology of Cognitive and Emotional Processes of the University of Guadalajara, Mexico by using a 128-channel Medicid (Neuronic) amplifier. However, like in the former cases, only the 19 standard channels from the 10/20 International System were recorded, employing an appropriate Electro Cap. The signals were referenced to the linked earlobes with filter set at 0.1–70 Hz and sampled at 200 Hz by using a neuronic acquisition program. All subjects were recorded while listening to an acoustic rhythm presented with different tempi (120, 140, and 160 bpm) with open eyes. The protocol was approved by the Ethics Committee of the Neuroscience Institute of the University of Guadalajara.

### 2.4. Definition of the Interrelation Measures

In order to measure relations between time series, we focus on two prominent quantities, namely the cross-correlation coefficient and the degree of phase synchronization estimated by the Mean Phase Coherence (Mormann et al., 2000). This choice is substantiated by the fact that none of the two measures require a computationally expensive and error-prone phase-space reconstruction such as with non-linear interdependence (Kreuz et al., 2007). As such a procedure is in general questionable for non-stationary, noise contaminated systems with a high dimensional phase space (Kantz and Schreiber, 2004). Furthermore, information theoretical measures also seems to be more affected by external noise and less sensitive for the detection of interrelationships between two signals. Because of its seemingly superior performance in EEG-analysis (Mormann et al., 2005), even in situations where coupled non-linear, low-dimensional systems are under consideration (Kreuz et al., 2007), we decided to restrict the bivariate analysis to linear cross-correlation coefficients and phase synchronization estimated by the mean phase coherence. From these bi-variate measures, symmetric interrelation matrices are constructed, which reflect the interrelations between all pairs of electrodes.

#### 2.4.1. The Zero-Lag Correlation Matrix

For a proper computation of the cross-correlation coefficients the *N* time series *X*_*i*_(*t*_*k*_) (*i* = 1, …, *N* and *k* = 1, …, *T*) are first normalized to zero mean and unit variance:

(9) $$\begin{array}{r} {\tilde{X}}_{i}\left(t_{k}\right)=\frac{X_{i}\left(t_{k}\right)-\langle X_{i}\rangle_{t_{k}}}{\sigma_{i}}, \end{array}$$

where brackets 〈*X*_*i*_〉 denote time average and σ_*i*_ the standard deviation estimated over a time window of length *T*. Then the *N* × *N* zero-lag cross-correlation matrix *C* is composed over the same data segment:

(10) $$\begin{array}{r} C_{ij}=\frac{1}{T}\overset{T}{\underset{k=1}{\sum }}{\tilde{X}}_{i}\left(t_{k}\right){\tilde{X}}_{j}\left(t_{k}\right)=\langle {\tilde{X}}_{i}\left(t_{k}\right){\tilde{X}}_{j}\left(t_{k}\right)\rangle_{t_{k}}. \end{array}$$

The matrix defined in Equation (10) is a real symmetric matrix and, being a quadratic form, it is also positive semi-definite.

#### 2.4.2. Phase Synchronization

The main deficiency of the zero-lag correlation coefficient consists in the fact that phase differences of π/2 cannot be detected by definition. The equal-time correlation between e.g., sine and cosine waves with the same frequency is zero, although there is a tight phase locking between the two signals. Therefore, in the present study we considered also a phase synchronization measure, which is sensitive for *any* phase differences between two signals and solely quantifies the stability of the phase locking of instantaneous phases over time.

A prerequisite for the characterization of phase synchronization is the proper estimation of the instantaneous phase ϕ(*t*), which is in fact a nontrivial issue (Picinbono, 1997; Chavez et al., 2006; Rios Herrera et al., 2016). In order to correctly assign a physical meaning to the ϕ(*t*), the signals have to fulfill certain requirements; in particular, they have to be narrow banded with only one prominent peak in the power spectrum (Chavez et al., 2006). Otherwise, band-pass filtering is mandatory (Rios Herrera et al., 2016).

If this condition is fulfilled, the instantaneous amplitude *a*(*t*_*k*_) and phase ϕ(*t*_*k*_) of a signal are then properly defined by the real and imaginary part of the analytic signal $\tilde{S}\left(t_{k}\right)=X\left(t_{k}\right)+iX_{H}\left(t_{k}\right)$ (Gabor, 1946; Oswald, 1956; Bedrosian, 1962):

(11) $$\begin{array}{r} a\left(t_{k}\right)=\sqrt{X{\left(t_{k}\right)}^{2}+X_{H}{\left(t_{k}\right)}^{2}}, \end{array}$$

(12) $$\begin{array}{r} \phi \left(t_{k}\right)=\arctan\left(X_{H}\left(t_{k}\right)/X\left(t_{k}\right)\right), \end{array}$$

whose imaginary part is obtained using the Hilbert transform:

(13) $$\begin{array}{r} X_{H}\left(t_{k}\right)=\frac{1}{\pi }\text{P}.\text{V}.\int_{-\infty }^{\infty }\frac{X\left(\tau \right)}{t_{k}-\tau }d\tau . \end{array}$$

Here P.V. denotes that the integral is taken in the sense of the Cauchy Principal Value. The temporal stability of possible phase relationships between signals *X*_*i*_(*t*_*k*_) and *X*_*j*_(*t*_*k*_) can then be estimated by

(14) $$\begin{array}{r} R_{ij}=\frac{1}{n}\left|\overset{n-1}{\underset{k=0}{\sum }}e^{i\Delta \phi_{ij}\left(k\Delta t\right)}\right|. \end{array}$$

In the last formula *k* denotes the time index, $\frac{1}{\Delta t}$ the recording frequency and *n* the length of the time interval used to estimate the ϕ(*t*_*k*_) and, hence, *R*_*ij*_ is an estimator for the temporal stability of the phase differences Δϕ_*ij*_(*kΔt*) = ϕ_*i*_(*t*_*k*_)−ϕ_*j*_(*t*_*k*_).

### 2.5. Evaluating Distortions

The main focus of the present contribution is to quantify distortions of the correlation structure induced by the reference signal. For this purpose, we calculated the average difference of the interrelation matrix obtained for the untransformed original data and its reference-transformed version:

(15) $$\begin{array}{r} D=\frac{2}{N\left(N-1\right)}\underset{i>j}{\sum }|M_{ij}^{Original}-M_{ij}^{Reference}|, \end{array}$$

where *N* is the number of simulated (or real) EEG channels. Note, in the above formula as well as in the sequel the symbol *M*_*ij*_ is used as a replacement for either the Correlation matrix *C*_*ij*_ or the Mean Phase Coherence matrix *R*_*ij*_, when formulas refer to interrelation matrices in a more general sense.

Eventually, we also calculate the average of the absolute values of the non-diagonal elements of the interrelation matrices:

(16) $$\begin{array}{r} <|M|>=\frac{2}{N\left(N-1\right)}\underset{i>j}{\sum }|M_{ij}|. \end{array}$$

In order to probe for possible distortions of univariate properties of the signals, we estimated the power spectra of the reference transformed signals and compared them with that estimated from the untransformed data. Eventually, we also calculated the power spectrum for the reference itself.

## 3. Results

We start this section with an exemplary discussion of one particular model, namely model 7 of Table 1, before we merge the results obtained for all models in a summary figure. Thereafter, we will consider the problem of re-referencing and probe the assumed independence of earlobe signals. Then we turn to possible deformation of power spectra and discuss probable dynamical changes due to a chosen EEG-reference as well as the influence of eye-artifacts at the end of this section. In all cases we provide a quantitative comparison of different EEG-reference schemes considered in the present study.

### 3.1. Deformation of Spatially Distributed Correlation Pattern

**Figure 1:**
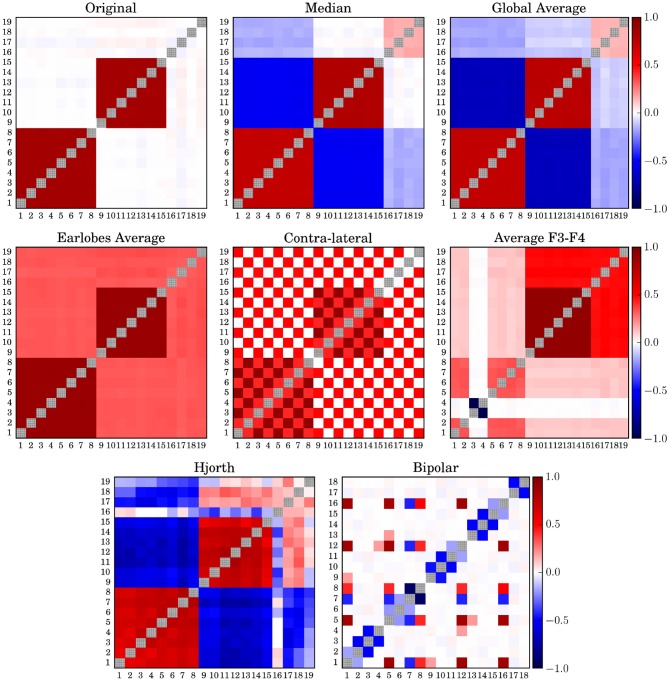
Average correlation matrices derived from model 7 for different simulated EEG references. The diagonal elements of all matrices are drawn in gray in order to improve visibility.

In order to study possible deformation of the spatial interrelation structure we derived time series from 2^14^ sampling points. Then we generated the zero-lag correlation matrix for a data window of 256-samples, which is equivalent to 1 s. Thereafter, we averaged over this set of correlation matrices. This procedure is done for the untransformed time series derived from *N*_*f*_-tori (7), as well as for the signals transformed to the above-mentioned reference schemes. Results for Model 7 are shown in Figure 1.

In order to improve the visibility of the spatial correlation structure of the matrices shown in Figure 1 all diagonal elements are shadowed gray. The average phase difference between the two clusters of model 7 is π/2 and, hence, by definition not detectable by zero-lag correlations. Therefore, the two clusters seem uncorrelated even for the case of the untransformed data. Median reference (M) and global average (gav) performed qualitatively similarly, as should be expected, although the distortions caused by the median were marginally less pronounced. This was probably due to the fact that the median is a non-parametric quantity, which is not vulnerable to outliers. However, both schemes induce erroneously anti-correlations between the two clusters while the earlobe reference, on the other hand, induces a pronounced positive offset over the whole matrix.

When the contra-lateral montage (CL) is used, which is to say when each hemisphere is referenced to the earlobe of the opposite hemisphere, a peculiar checkered pattern of spurious correlation distributed over the whole matrix, while at the same time certain genuine intra-cluster correlations become significantly weakened. Major distortions of the spatial pattern are also observed for the (*F*3*F*4) as well as for the bi-polar reference (bp). In both cases a massive destruction of genuine correlations as well as the generation of spurious correlations takes place, such that the interpretation of the spatial correlation pattern as the functional brain network would lead to completely erroneous conclusions. In comparison, the functional network derived in the Hjorth-montage (H) is much closer to the untransformed one, although in that case the truly uncorrelated data channels 17–19 seem to be strongly correlated or anti-correlated to both correlation clusters, in addition to pronounced but mistaken anti-intercluster correlations.

**Figure 2:**
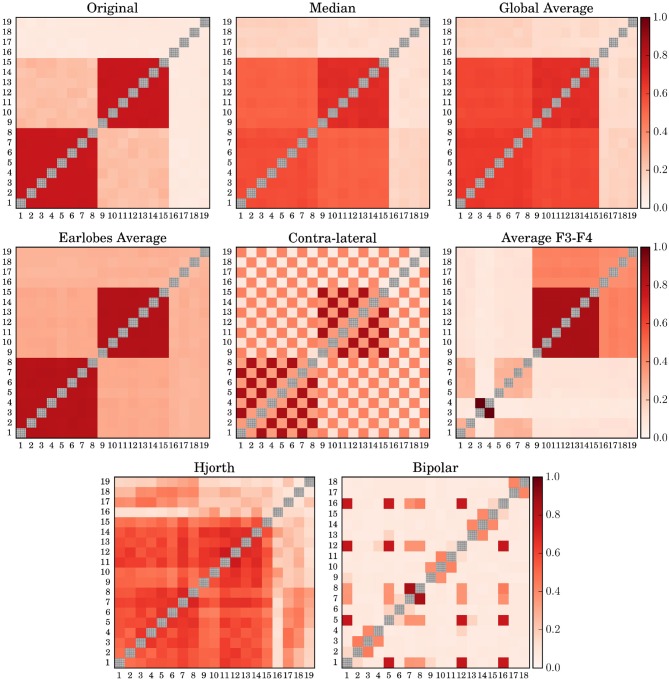
Average Mean Phase Coherence matrices derived for model 7 by using different simulated EEG references. The diagonal elements of all matrices are drawn in gray in order to improve visibility.

When turning to phase synchronization estimated by the mean phase coherence (Figure 2), one observes a similar picture. Major distortions due to the choice of the EEG-reference are observed for the (*F*3*F*4)-reference, the contra-lateral scheme and the clinically relevant bipolar montage. For phase synchronization the Hjorth-reference also performed worse, given that for model 7 even the two clusters with a mean phase difference of π/2 could not be identified but seemed to merge into a single large group of correlated channels. Like in the case of cross-correlation, the earlobe reference generated an artificial bias, such that the inter-cluster interrelation gets somewhat hidden by this offset. Median reference and the global average are quite similar as expected, with slightly better results for the usage of (M). For both schemes, the genuine intra-cluster synchronization strength is somewhat weakened. However, the two cluster configuration as well as the inter-cluster synchronization are clearly visible.

**Figure 3:**
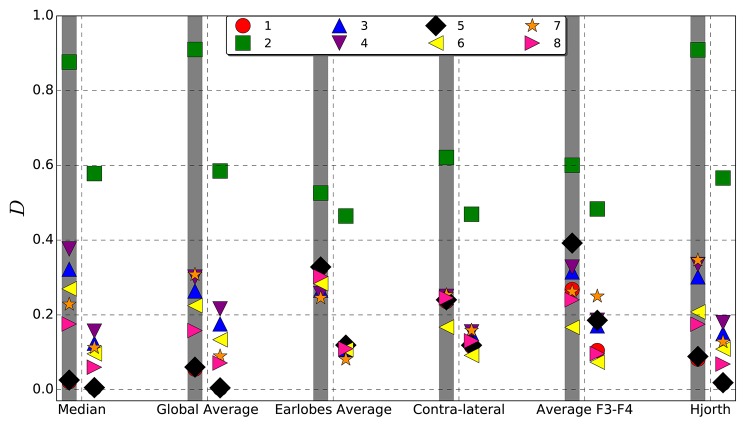
Average deviation *D* (Equation 15) from the original, viz. untransformed interrelation pattern caused by different reference schemes estimated for synthetic data derived from each of the models listed in Table 1. Left columns, indicated by a gray shadow, show the results obtained for the correlation matrix, right columns those for the mean phase coherence matrix.

To quantify the difference between the interrelation pattern derived for the untransformed data of the models listed in Table 1 and the reference transformed signals, we employ Equation (15). Note, with the set of models listed in Table 1 we intend to include qualitatively different spatial interrelation patterns beginning with no correlations at all, one large cluster as well as multi-cluster configurations. In order to obtain reliable results we created 100 realizations for all models. In each case we estimated the cross-correlation matrix as well as the mean phase coherence matrix over a data segment of 4,096 data points. In order to avoid edge effects, 10% at each side of the segment is disregarded (Mormann et al., 2000). For both bi-variate measures symmetric interrelation matrices were constructed, which reflect the interrelations between all pairs of electrodes. Then, deviations were estimated according to Equation (15). The results summarized in Figure 3 allow a quantitative comparison of the reference schemes considered.

In general, one observes that deviations from the original pattern are less pronounced when the mean phase coherence is estimated in order to construct the functional network. Deviations for linear correlations are systematically larger. However, qualitatively the results for both estimators reflect similar features. Namely, for Model 2, where all data channels are correlated, the deviations are most pronounced for all reference schemes, but the largest values are observed for the median, the global average and the Hjorth transformation. Also for Model 4, where more than the half of the signals are correlated, the median reference shows the worst performance. This could be expected given that the median as well as the global average is dominated by correlated signals if interrelation clusters are large. In that case, cluster-specific features might be subtracted from correlated channels and artificially induced to independent signals.

On the other hand, this effect might also have positive consequences, namely concerning the undesired contamination via volume conduction. Hereby, redundant information is induced to all active electrodes, such that the corresponding signals are no more exclusively reflecting neuronal activity of the vicinity of each electrode. Hence, by volume conduction additional spurious zero-lag cross-correlations are generated. However, by re-referencing to the global average as well as to the median reference, redundant information is subtracted. Therefore, the deviations observed for Model 2 in Figure 3 are due to the fact that the global correlation pattern covering all active electrodes is eliminated by re-referencing to (gav) and (M) and, in this case, the data looks like the completely uncorrelated recordings of Model 1, where both references perform best. Yet, when correlation structures are small, or when multiple correlation clusters are present, deviations caused by the median reference as well as the global average are the least.

In contrast, the deviations obtained for the earlobe reference seem to be almost independent from the correlation structure of the data set. Quantitatively, as documented in Figure 3, the overall distortions of the earlobe reference are approximately of the same order of magnitude as those observed for the contra-lateral scheme. For all models considered in the present study we obtained almost the same moderate amount of distortions for both the mean phase coherence and the correlation matrix for the case of the earlobes average. However, this observation is owed to the fact that we assumed in this model calculations that the earlobe signals are completely independent from those measured by the scalp electrodes. In this case only a constant bias, as observed in Figures 1, 2, can be expected and of course, by construction this bias does not depend on the genuine spatial correlation pattern.

Nonetheless, it is plausible to assume that in a true measurement, the earlobe signals share information with (at least some) active electrodes. If that is true, the deviation from the true correlation pattern is not just a constant, spatial homogeneous offset, but one should expect a more complicated distortion, such that genuine correlations could be destroyed in one region while spurious correlations could be generated in another, like in the case of the other reference schemes. We probe the independence of earlobe signals in the next section.

Slightly more scattered are the results obtained for (*F*3*F*4) and (*H*), showing deviations of the same magnitude as those estimated for (*M*). However, these reference schemes provoke severe structural changes, which are not capable of the global measure 15 shown in Figure 3 but clearly documented by the example of Model 7 in Figures 1, 2.

### 3.2. Independency of Earlobe Signals

In order to probe for a possible dependency on earlobe electrodes, we consider the whole night EEG recording of a clinically healthy subject, with A2 as the reference electrode (Dataset 1 of section 2.3). In addition to the common 19 active channels of the 10/20-system, the left earlobe A1 was also recorded. Hence, in this case we were able to estimate the cross-correlation of A1 with each of the active ones.

**Figure 4:**
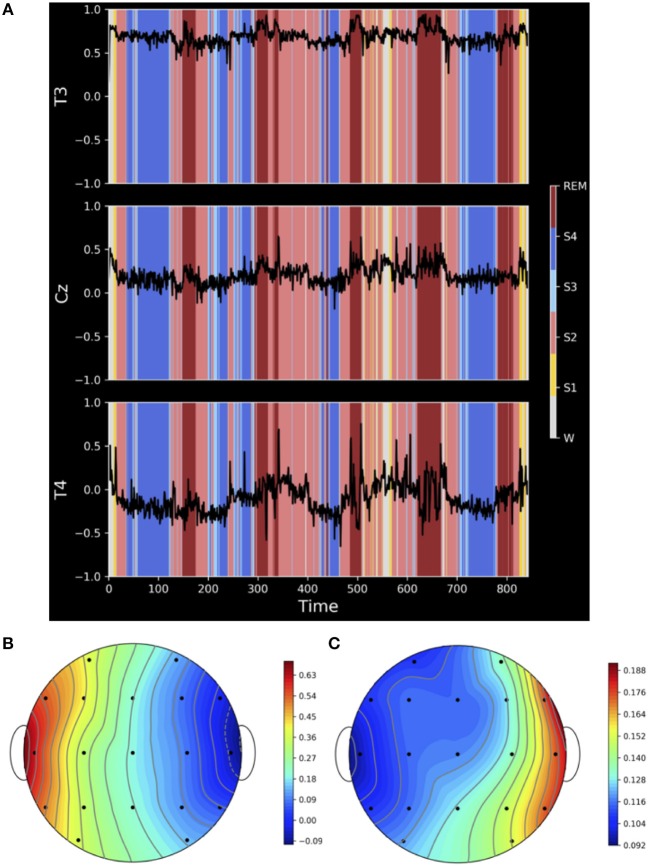
**(A)** Correlation coefficient between the earlobe A1 and active electrodes T3, Cz and T4 estimated with a running window of length 3,840 data points (corresponding to 30 s) over the whole night recording of a clinically healthy, 26-year-old male subject. Sleep stages (W = awake, S1–S4 denotes sleep stages 1–4 and REM denotes Rapid Eye Movement sleep periods) are indicated by colored shadows in each panel. **(B)** Shows a heat map of the average correlation between the active electrodes and A1 and **(C)** the respective standard deviation. Both quantities are estimated over the whole night recording.

The results displayed in Figure 4 document that, despite our model assumptions, strong correlations between the earlobe and active electrodes are present, e.g., in the case of electrode T3 (Figure 4A). Additionally, correlations between earlobe and active electrodes may vary strongly with the physiological brain state and may even change its sign. For instance, electrode T4 shows a pronounced dependency on sleep stages and vary notably during sleep stage 2, deep sleep and awake state. In fact, all active electrodes are correlated with A1 with highest average values for electrodes located on the same frontal or temporal lobe like F7 (*C*_*av*_ = 0.58), T3 (*C*_*av*_ = 0.68) and T5 (*C*_*av*_ = 0.6). On the other hand, electrodes located at the right temporal lobe, like F8 (*C*_*av*_ = −0.07), T4 (*C*_*av*_ = −0.1) and T6 (*C*_*av*_ = −0.03), are on the average weakly correlated with A1, but those correlations may show a pronounced time dependence as exemplified in Figure 4. Finally, average correlations at the midline like Fz (*C*_*av*_ = 0.2), Cz (*C*_*av*_ = 0.18) and Pz (*C*_*av*_ = 0.2) are still not negligible. The heat maps in the lower part of Figure 4 illustrate nicely that the strength of those correlations decrease with the distance to the earlobe (Figure 4B), while simultaneously fluctuations, caused by the time dependency of the interrelation between earlobe and active electrodes and, hence, the dependency on physiological brain states, notably increase (Figure 4C).

Based on these findings we conclude that the assumed independence of earlobes in our theoretical model is unrealistic for EEG recordings and favors notably this reference. Hence, results presented for the earlobes average in Figure 3 are just estimates of minimal possible average deviations and, due to the fact that correlations vary across the scalp and may strongly vary in time, one has to expect that the earlobe reference causes, additionally to the observed bias, important spatiotemporal deformations of the genuine interrelation pattern, which additionally affect notably the time evolution of the measured functional network due to its marked dependency on the physiological brain state.

### 3.3. Re-referencing to the Median Reference

**Figure 5:**
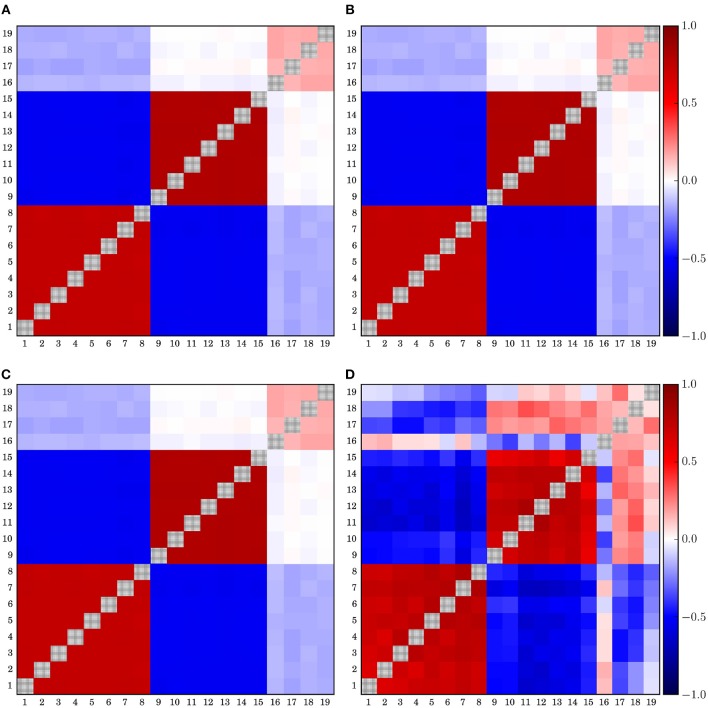
Correlation matrices estimated for median-referenced data previously transformed to different popular EEG-references. **(A)** A1A2, **(B)** global average, **(C)** F3F4, and **(D)** Hjorth. Data have been derived from Model 7 of Table 1, like in Figures 1, 2.

According to the results presented so far, and considering that synchronous dynamics of a large number of electrodes occurs mainly under pathological circumstances like generalized epileptic seizures, it seems, according to the results presented in Figure 3 and considering the discussion made in section 3.2, that the median reference and the global average performs best under quite generic conditions, if the construction of the functional network of the electrical brain activity is desired and no strong global correlations covering most of the active electrodes are to be expected. However, EEG-equipment do not allow for data recording using these reference schemes. Thus, both references might depend on the reference scheme used during data acquisition and so we tested in the next step for such a potential dependency. In Figure 5 we summarize the corresponding results obtained for artificial data derived from Model 7 (like in Figures 1, 2) for the median reference. Equivalent results were obtained for the global average.

The correlation matrix conserves even quantitatively the same structure when the data are retransformed from the global average Figure 5B. Given that the global average is usually close to the median if the sample does not contain crucial outliers or a pronounced asymmetric tail, this result could be anticipated. However, the same is true when the data is at first transformed to the average of two active electrodes (F3F4) (Figure 5C) or the average of the earlobe electrodes (A1A2) (Figure 5A). Also for these cases, one recuperates not only qualitatively the same correlation structure, but quantitatively the obtained correlation matrices are also remarkable similar. On the other hand, the correlation matrix derived from data previously transformed to the Hjorth-reference is markedly different (Figure 5D). In this case, one retrieves the correlation structure already seen in Figure 1, i.e., the re-transformation to the median does not provoke notable changes, such that the deformations caused by the Hjorth-transform are almost conserved. Due to the fact that in the Hjorth montage to each electrode a personal reference signal (determined by its neighborhood) is assigned, signatures imprinted by the Hjorth transform can no more be eliminated by re-referencing. Similar results are obtained when the mean phase coherence is used instead of cross-correlations (not shown in the figure). In conclusion, these results underpin the robustness of the median reference (as well as the global average) against re-referencing. The resulting interrelation matrices are practically independent from the EEG reference used during the recordings. This is particularly true for the commonly used earlobe reference, mastoids or when an active electrode is employed as a reference point.

### 3.4. Influence on Power Spectra

**Figure 6:**
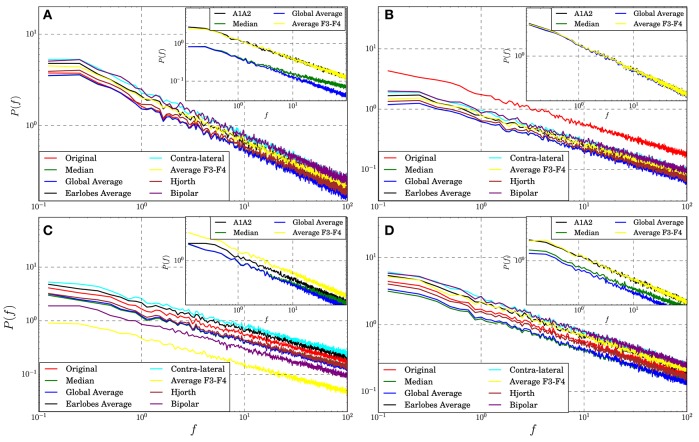
Power spectra averaged over all active electrode signals transformed to different reference schemes estimated for synthetic data derived from Model 1 **(A)**, Model 2 **(B)**, Model 7 **(C)**, and Model 8 **(D)**. The insets show the power spectra of some of the corresponding reference signals.

So far it remains unclear if and to what extent EEG-references may influence univariate properties of the recordings. Here we test for linear autocorrelations expressed by the power spectra of the transformed data in comparison to those of the untransformed signals derived from the theoretical models. Note, the power spectrum is just the Fourier transform of the autocorrelation function and thus, its slope is a direct measure of the autocorrelation length. This procedure seems to be reasonable given that reference transformations are linear and as such they may modify linear univariate properties. In Figure 6 we show the mean power spectra of an active electrode, averaged over an ensemble of 100 realizations of four different models. Additionally, the power spectra of the reference signals are drawn in the respective insets.

For the model data we observe for all cases that autocorrelations are almost conserved. For all models and all reference schemes considered in the present study the slopes of the power spectra do not vary notably from that of the untransformed data. Slope variations are tiny and only the absolute power gets modified, although in some cases the autocorrelation length of some reference signals differs. For example, the slope of the power spectrum of the median reference derived for Model 1 (inset of panel Figure 6A) is somewhat lower than that of other reference signals, indicating a shorter auto-correlation length. Nevertheless, on the average, linear univariate properties expressed by the power spectra seem to be quite robust under reference transformations, and they are at least for the test framework considered in this study only marginally affected.

### 3.5. Applications to Real World Data

Figure 4 already indicates that correlations induced by the EEG reference are by no means stable in time, but may depend crucially on the genuine correlation pattern of the specific physiological brain state. This is explicitly exemplified for the case of the earlobe reference in section 3.2. There we could prove that the correlations between the earlobe signals and active electrodes not only depend on the distance to the earlobe but show also notable variations with the sleep stages. Here we further investigate this issue by using: (a) a real EEG recording of an epileptic seizure, (b) a sleep recording of a healthy subject, and (c) recordings of healthy subjects with open eyes while hearing a rhythmic acoustic stimuli. To this end we proceed in three steps. First we show that the time evolution of the overall correlation strength during the peri-ictal transition of an epileptic seizure may differ qualitatively between different reference schemes. However, given that the true dynamics of the functional network is effectively unknown for real world recordings, we use in the next step the sleep recording solely for the deduction of reference signals, which are then applied to artificial data of Model 1, where all data channels are uncorrelated. This allows a more objective, quantitative evaluation of disturbances caused by the different reference schemes. Finally, in a last step we use the data sets of healthy subjects in order to test for robustness against muscle artifacts, in particular eyelid movement.

#### 3.5.1. Correlation Dynamics of a Focal Onset Seizure

**Figure 7:**
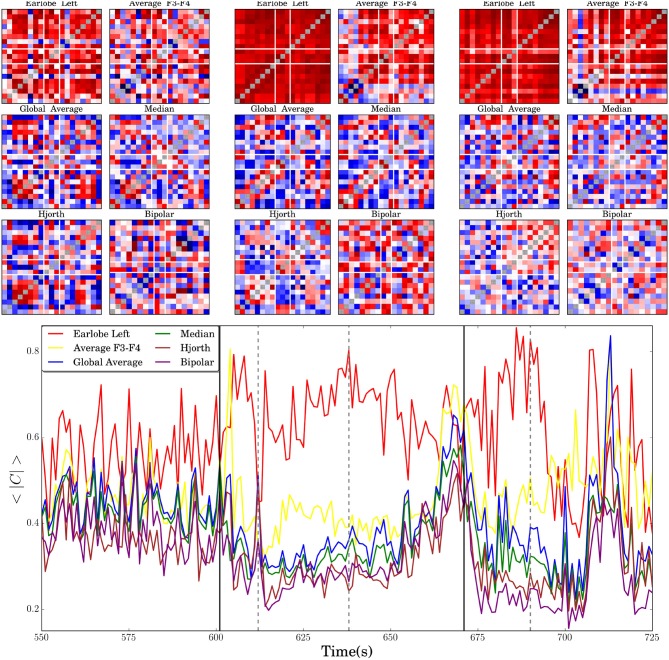
In the lower panel the time evolution of the average absolute value of the non-diagonal elements of the correlation matrices 〈|*C*|〉, estimated for an extracranial recording of a focal onset seizure, is shown. Vertical black solid lines indicate seizure onset and offset. Dashed vertical lines mark three instances of time for which the cross-correlation matrices, obtained for different reference montages, are shown in the upper part of the figure. The first two columns of six matrices correspond to the moment just after seizure onset (around second 610), the next two columns to the instant marked in the central part of the seizure (about second 635) and the matrices of the last two columns are estimated for the marked instant of time during the post-seizure period (about second 690). In all cases the diagonal elements are artificially set to gray color in order to improve visibility.

In this section we present the results for the analysis of the time evolution of a spatial cross-correlation pattern derived from an extracranial EEG-recording containing a focal onset seizure. Due to the fact that we consider here empirical data recorded from a real world experiment, the genuine interrelation pattern of the multivariate data set is unknown. Therefore, in this section we are only able to compare qualitative differences of the time course of numerical estimates. In Figure 7 we show in the bottom the average absolute cross-correlation coefficient derived for recordings transformed to different reference schemes along the peri-ictal transition of the seizure. In the same figure we show additionally cross-correlation matrices obtained for different reference schemes in order to exemplarily visualize the variety of spatial interrelation patterns that may occur in the different settings.

Figure 7 nicely documents that the influence of EEG-references is not necessarily stationary but may alter qualitatively the time evolution of the correlation pattern. 〈|*C*|〉(*t*) takes almost always the highest values for the left earlobe reference, which is in line with the findings reported above. The redundant information induced by the earlobe signal enhances notably the overall correlation strength and, hence, it is expected that values obtained for 〈|*C*|〉(*t*) are larger than those obtained for other reference montages. Furthermore, temporal changes of 〈|*C*|〉(*t*) are notably diminished in comparison to the curves obtained for other references, such that even the ictal event is only hardly discernible in the time evolution of the average correlation strength. Apparently, the bias induced by A1 overshadows the correlation matrix and dynamical features of the brain activity get markedly obscured.

In addition, it seems that distortions of the interrelation pattern, probably due to the fact that the earlobe is not independent from the active electrodes, are expressed by a pronounced anti-correlation between the results derived for the *A*1-reference in comparison to others. This is particularly true for the period between second 600 and 700. During the seizure 〈|*C*|〉(*t*) is somewhat elevated, it decreases toward seizure offset and rises again in the immediate postseizure period. On the contrary, the time course of 〈|*C*|〉(*t*) derived for the remaining reference schemes shows a pronounced minimum during seizure and a strong increase toward seizure offset. Then it encounters again a local minimum during the immediate postseizure period. Only when two active electrodes are used (*F*3*F*4) does one observe quantitative and occasionally also qualitative deviations from the behavior revealed for the median, global average, the Hjorth and the bipolar montage.

Again, we underline that the time evolution of the genuine correlation pattern of real world data is unknown. However, the loss of correlations during seizure and the strong increase toward seizure offset are in accordance with previous findings for intracranial recordings (Schindler et al., 2006), using eigenvalues as an indicator for the overall increase (or decrease) of cross-correlations within the multivariate dataset. In this work, the excess of correlations is not understood as the pathology but as an active mechanism used by the brain in order to terminate the crisis.

〈|*C*|〉(*t*) provides solely information about the time evolution of the overall correlation strength. Possible spatial deformations are not captured by this quantity. The correlation matrices shown at the top of Figure 7 exemplify spatial deformations and confirm various conclusions drawn from the analysis of the model data presented above. One observes clearly that the cross-correlations obtained for the (A1)-reference are notably elevated in comparison to the matrices derived for the other reference schemes. For example, the correlation matrix estimated in the central part of the seizure is quite similar to the matrix drawn for the post-seizure period. The remarkable offset blurs the spatial interrelation pattern and, given that the earlobes are not independent from the signals recorded from the active electrodes (see Figure 4), it gets additionally distorted. Furthermore, and on contrary to the time evolution of 〈|*C*|〉(*t*), one observes that the correlation matrices obtained for the remaining reference schemes show qualitatively different interrelation patterns, with the exception of the median and the global average, whose matrices are remarkable similar.

In any case, the correlation matrices reveal not only an increase or decrease of the overall correlation strength but, furthermore, one encounters structural changes of the interrelation pattern during the ictal and post-ictal period. The three matrices obtained for *F*3*F*4, bipolar scheme and Hjorth transform show drastic structural changes along the peri-ictal transition. This behavior indicates a high sensitivity to physiological changes, probably the reason why the bipolar montage is so convenient for clinical purposes. For the bipolar and Hjorth transform the reference is defined by the neighborhood of an active electrode, viz. this observation indicates that the transition from ictal to postictal activity is accompanied by pronounced local changes of inter-dependencies. However, according to the results presented above in Figures 1–3, neither of these schemes is adequate for the study of the functional network due to drastic deformations of the genuine spatial interrelation pattern.

#### 3.5.2. Deep Sleep and Paradoxical Sleep

Here we consider a 19-channel sleep EEG of a healthy subject (data set 1 of section 2.3) and focus on deep sleep (sleep stage 4) and periods with Rapid Eye Movement (REM-sleep). Sleep stage 4 is particularly characterized by large amplitude slow oscillations with a higher level of synchronization between brain regions (Guevara et al., 1995). In this sense, at least for the slow frequency band one expects a more collective dynamical state. REM-sleep, on the other hand, is characterized by a larger contribution of fast frequencies, and the morphology of the EEG-signals is more similar to the awake state.

In order to visualize dynamical changes we derive different reference signals from EEG segments of both sleep stages for the δ− and the fast β−band. These reference signals are then used to transform synthetic data derived from Model 1 of Table 1, of 19 independent signals simulating the active electrodes. Hence, in this model no genuine cross-correlations are present, but, as in the previous section, each synthesized data channel is assigned to a specific electrode of the 10/20 system in order to apply the reference schemes such as e.g., the bi-polar or the Hjorth setting.

**Figure 8:**
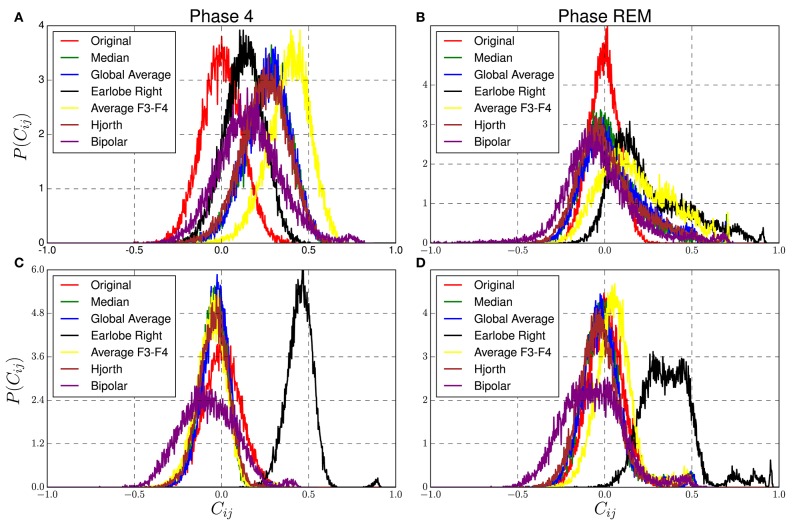
Distribution of non-diagonal elements of correlation matrices estimated from reference transformed data derived from model 1 of Table 1. Reference signals are extracted from real EEG-recordings of a healthy subject during sleep stage 4 **(A,C)** and REM sleep **(B,D)**, for the δ−band **(A,B)** and the fast β−band **(C,D)** as described in the main text.

For example, in the case of the bipolar reference the EEG recorded at *F*7 was used as a reference for data channel *Fp*1 of the model data, *T*3 of the real EEG as a reference for data channel *F*7 of the model data, etc. For the global average and the median reference, the corresponding references have been derived from the real EEG in order to re-reference the uncorrelated model data. That is to say, separately for the fast and slow frequency band as well as for the deep and paradoxical sleep each reference has been derived from real world recordings in order to re-reference uncorrelated artificial data. In order to create a realistic situation, we normalized the model data such that the standard deviation of the signals coincides with those of the corresponding EEG recordings. Note, we do not claim that the real world data, used to derive the reference signals, do not contain genuine correlations. But with the exceptions of the bipolar and the Hjorth montage, these correlations do not play any role in the present context, such that the reference solely induces the same redundant information to each signal of the model data. If, on the other hand, each electrode receives its own reference given by a single electrode or the neighborhood of some electrodes, genuine correlations of the EEG recordings are partly transmitted to the formerly uncorrelated synthetic data. But as we can see below, they play only a minor role for the present analysis. Figure 8 shows the distribution of non-diagonal elements for the original and reference transformed data for both sleep stages in the two frequency bands.

Due to the lack of genuine correlations, the nondiagonal elements of the cross-correlation matrices derived from Model 1 should be equal to zero, but because of the existence of random correlations one encounters for the untransformed data a symmetric distribution centered at zero. For the reference transformed data it is desired that corresponding distributions are as close as possible to this null-hypothesis. However, one observes clearly that conspicuous deviations are present for all reference schemes, which depend on both the physiological brain state as well as the frequency band.

The worst performance is observed for the earlobe reference during REM-sleep for both frequency bands and for the β−band during deep sleep. It turns out that the above mentioned bias of this reference scheme is larger for fast than for slow frequency bands for both sleep stages (see Figures 8B–D). In the fast β−band, the corresponding distribution is notably displaced toward considerably large correlation values around 0.5, but in particular for REM-Sleep a long tail with values close to one is observed. Note, these deviations are not caused by variations of the power in the delta or beta-band of the model data but are due to the reference transformation. Variation of the relative power would be equivalent to the variation of the amount of random correlations. In this case, one should not expect any displacement but just a broadening of the probability distributions (Müller et al., 2011). Variations of the relative power do not shift the center of the distribution functions toward lower or larger values but just vary its standard deviation. Displacements, on the other hand, are caused by the induction of redundant information to all data channels, because it increases the correlations between all data channels of the model data almost equally. If in addition the EEG data are non-stationary, such that the standard deviation of the reference alters with time, also higher moments of the probability distributions get affected, because then the center of the distribution does not stay at a constant value, and fat tails, as observed in Figure 8, are generated. Here we used a considerably long segment of the sleep stage 4 of the first sleep cycle and two data segments of REM sleep taken from different sleep cycles. In both cases the stationary assumption is presumably no more valid (Dijk et al., 1990).

Hence, the poor performance in the fast frequency bands can be explained by the ratio of the standard deviation of the earlobe signal and that of the active electrodes, which is directly related to the amount of redundant information induced by the reference. For sleep stage 4 in the delta band this ratio is 0.38, viz., the magnitude of the earlobe signal is considerably smaller than that of scalp electrodes. Correspondingly, the shift toward larger correlation values is moderate. For REM sleep in the delta band this ratio is with 0.76 notably larger, and a shift toward larger mean correlation values and additionally a fat tail toward large correlations is generated (see Figure 8B). However, for the fast frequency band, these ratios are 0.92 and 0.93, respectively, for deep and REM sleep. In these cases, the redundant information imposed by the reference is of the same order of magnitude as that of the simulated EEG-signals themselves. Consequently, the distributions are located close to 0.5 (see Figures 8C,D).

On the other hand, when slow waves during deep sleep are considered, the earlobe reference shows smallest deviations and clearly outperforms the other settings. Given that in particular the slow frequencies in the δ−range constitute a kind of collective dynamics, a major influence on the global average and median reference is expected. Correspondingly, the distributions derived for the global average and the median reference (as well as for Hjorth transform) are centered approximately at 0.36. On the contrary, for the slow waves during REM-sleep (Figure 8B), the median reference and global average are closest to the null hypothesis. The same is true for the β−band for both sleep stages, where deviations obtained for the median and the global average montage are smallest.

Finally, also the bipolar and Hjorth montage show a notably good performance, given that the corresponding probability distributions are considerably close to the null-hypothesis of the untransformed model data. However, as mentioned in the last section, crucial deformations of the topological properties of the functional network caused by these references disqualify them for connectivity studies.

#### 3.5.3. Vulnerability to Artifacts

**Figure 9:**
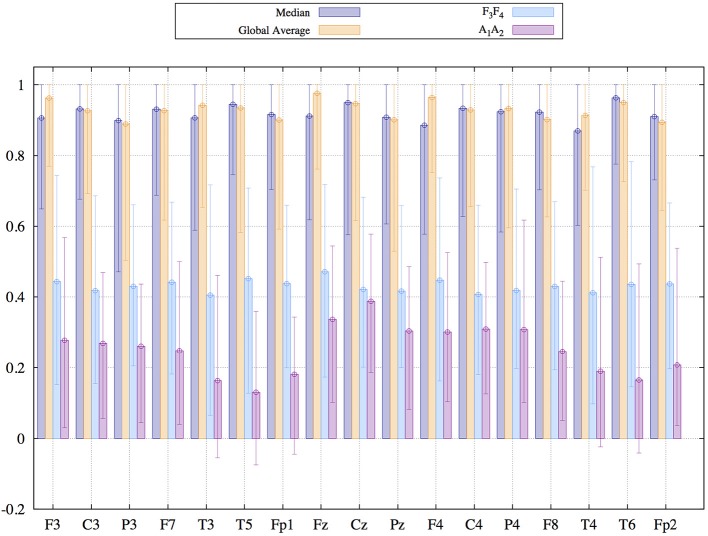
Median and 95% confidence interval of the Pearson coefficient comparing signals recorded by the same electrode before and after the application of Independent Component Analysis for the elimination of eyelid-movement. EEG recordings stem from data set three described in the method section.

As a final aspect of the present study we focus on the susceptibility to deformations due to artifacts, in particular to eye-artifacts. To this end we focus on EEGs from data set 3, which has been measured with respect to the linked earlobes reference, as described in the method section 2.3. For each reference scheme considered in this section, we generated two data sets. The first one was just the transformation to the new EEG-reference. For the generation of the second one, the original recordings have been preprocessed in order to diminish the influence of eyelid movements via the application of Independent Component Analysis (ICA). Then, after this pre-processing procedure, the cleaned data sets were transformed to different reference schemes as well. Thereafter, we estimated the Pearson coefficient between the cleaned and the raw data for each reference scheme separately. If the similarity between preprocessed and raw data is high (large Pearson correlation), the time-consuming pre-processing via the application of ICA is of minor importance. In such cases the transformation to the new EEG reference simultaneously eliminates to a large extent the contamination with artifacts. Otherwise, if the correction of artifacts causes serious morphological changes, which are not attained by the reference transformation, the Pearson correlation should be small. Results for all active scalp electrodes are shown in Figure 9.

Again, global average and Median reference lead to similar results. In both cases the central values of the samples of the Pearson coefficients are about 0.9, an extremely high value. This result implies that corrections effectuated by the ICA are insignificant such that the raw signals and the corrected ones are highly similar. We mentioned already above that events common in all signals get diminished by these references, depending on the collectivity of the episode, which has a quite positive influence with respect to the undesired effect of volume conduction, as outlined in the discussion of Figure 3. It seems that eyelid movements affect a sufficient number of signals, such that a posterior ICA only causes marginal corrections after transforming to the global average or the median. Simultaneously, correlation coefficients obtained for the earlobe reference vary between 0.1 for temporal electrodes and 0.4 for electrodes located at the central line. In this case, ICA is able to accomplish major changes, probably improving notably the signal quality. In other words, as a favorable side product the median reference and global average auto-correct to a high degree undesired collective artifacts. Time-consuming pre-processing of the recordings via ICA is of minor importance. On the other hand, when using the earlobe reference or active electrodes like *F*3*F*4, the application of ICA might be mandatory.

## 4. Summary and Conclusions

In general, the EEG-reference causes distortions of the spatial interrelation pattern in real world data, independently of which reference scheme is used (Figures 1–3 as well as Figure 7). Genuine correlations may get obscured, correlations may change the sign, or formerly uncorrelated channels may get (anti-)correlated to others. However, different reference schemes have different effects on the interrelation pattern, while autocorrelations expressed by power spectra are seemingly conserved, as revealed in the present study (Figure 6).

According to our model calculations, the earlobe reference causes an offset to all elements of the interrelation matrix independently of whether phase synchronization or cross-correlations are under consideration (Figure 3). This is due to the fact that the earlobe signal is induced to the recordings of all active electrodes (Figures 1, 2) and consequently adds redundant information to them. Therefore, independent from the genuine correlation pattern, the magnitude of the distortions takes always a moderate value (Figure 3). However, in these model calculations we assumed that the electrical potential at the earlobes is independent from the electrical activity measured upon the scalp—an assumption which is not accomplished as we could demonstrate via the results displayed in Figure 4. Depending on the distance to the earlobe, the correlation with an active electrode might encounter surprisingly high values of up to 0.75 or higher and, furthermore, these correlations show also dynamical changes depending on the physiological brain state. Consequently, our estimates based on model calculations for the earlobe reference are the most optimistic ones and, beside the pronounced bias, one should expect additionally major distortions of the spatial interrelation pattern. This is possibly the reason for the marked difference in the time evolution of the overall correlations during an epileptic seizure in comparison to other reference schemes, as exemplified in Figure 7. A strong bias elevates notably the curve obtained for the earlobe reference and simultaneously diminishes physiological changes of the correlations. But additionally, due to the distance-dependent correlations between the earlobes and scalp electrodes (see Figure 4), the topology of the functional network gets deformed. This is also documented in Figure 8, where reference signals have been derived from real world recordings. The strong dependency of the earlobe signals on the physiological brain state, and therefore their influence on the time evolution of the estimated functional network, remains evident. For the earlobe signal, the worst performance is observed in most situations displayed in Figure 8.

The performance of the median reference as well as the global average depend also on the physiological brain state. When several small or moderate interrelation clusters are present, both schemes show best performance. As shown in Figure 3, overall deviations caused by these references are minimal in most cases. Only when huge interrelation clusters are formed, imitating hypersynchronized brain activity involving almost all scalp electrodes, deviations are of the size (or larger) of those observed for the other reference schemes. In such situations both the median as well as the global average subduct a large part of the correlation strength of the large cluster and, simultaneously, they add artificially correlations to the remaining data channels. However, such hypersynchronized activity is probably not the generic case and occurs solely in particular circumstances. Additionally, this undesired effect might be beneficial in another context, namely the correction of muscle artifacts.

As shown in Figure 9 ICA-corrected and non-corrected signals are quantitatively notably similar. This result indicates that eyelid movements are automatically reduced by the reference transformation, an effect owed to the high collectivity of such events. In any case, Figure 9 implies that a time-consuming correction procedure like ICA is no more necessary when median or global average is used as a reference frame.

Finally, for all schemes using active electrodes as a reference, major distortions of the functional network are observed. This is true for the average of the electrodes F3 and F4 (and equivalently when Cz is used instead), as well as for the Hjorth transform of the bipolar montage. Furthermore, the contralateral reference scheme (as well as the ipsi-lateral scheme) causes a peculiar distortion perturbing the whole functional network (see Figures 1, 2). In all these settings the functional network gets almost completely blurred, which makes them unacceptable for the construction of functional networks.

However, we like to underline that these results are only relevant if one aims to construct the functional brain network from EEG recordings by using popular bi-variate measures like the mean phase coherence or linear cross-correlations. If one has other applications in mind, such schemes might be useful and one should prove the utility of a certain reference schemes for each case separately. For instance, for a clinical evaluation of epilepsy patients, the bipolar montage is well established and for multiple reasons the most useful one, although in this scheme the functional network is blurred almost completely. In this respect, and probably for most of the physiological brain states, the median reference as well as global average seem to deliver the most reasonable results. However, the optimal choice of the reference site depends on the particular study and on the purpose of the analysis as already stated in Dien (1998).

## Data Availability

EEG datasets are available to interested researchers, contact the corresponding author for requests.

## Ethics Statement

Sleep data measured in the Faculty of Psychology of the UNAM: This study was carried out in accordance with the recommendations of the Declaration of Helsinki (1964) with written informed consent from all subjects. All subjects gave written informed consent in accordance with the Declaration of Helsinki. The protocol was approved by the Ethical Committee of the Faculty of Medicine of the Universidad Nacional Autónoma de México.

EEG of the epileptic patient from the Clinical Research Neurological Institute of Colombia: This study was carried out in accordance with the recommendations of the Declaration of Helsinki (1964) with written informed consent from all subjects. All subjects gave written informed consent in accordance with the Declaration of Helsinki. The protocol was approved by the Ethics Committee of the Clinical Research Neurological Institute of Colombia.

Resting state EEG of healthy subjects measured in the Institute of Neuroscience in Guadalajara: This study was carried out in accordance with the recommendations of the Declaration of Helsinki (1964) with written informed consent from all subjects. All subjects gave written informed consent in accordance with the Declaration of Helsinki. The protocol was approved by the Ethical Committee of the Faculty of Psychology of the University of Guadalajara, Mexico.

## Author Contributions

MC-C, JR-L, and JZ-B provided the EEG-data. MM and WR-H designed the research. WR-H and PO-R wrote the code. WR-H and PO-R analyzed the data and produced the figures. MM wrote the manuscript. All authors participated in the discussion of the results, reviewed, and approved the final version of the paper.

### Conflict of Interest Statement

The authors declare that the research was conducted in the absence of any commercial or financial relationships that could be construed as a potential conflict of interest.

## References

[B1] Acar Z. A. Makeig S. (2013). Effects of forward model errors on EEG source localization. Brain Topogr. 26, 378–396. 10.1007/s10548-012-0274-6 23355112 PMC3683142

[B2] Alarcon G. Guy C. Binnie C. Walker S. Elwes R. Polkey C. (1994). Intracerebral propagation of interictal activity in partial epilepsy: implications for source localisation. J. Neurol. Neurosurg. Psychiatry 57, 435–449. 10.1136/jnnp.57.4.435 8163992 PMC1072872

[B3] Bedrosian E. (1962). The analytic signal representation of modulated waveforms. Proc. IRE 50, 2071–2076. 10.1109/JRPROC.1962.288236

[B4] Bertrand O. Perrin F. Pernier J. (1985). A theoretical justification of the average reference in topographic evoked potential studies. Electroencephalogr. Clin. Neurophysiol. 62, 462–464. 10.1016/0168-5597(85)90058-9 2415344

[B5] Brown R. E. Basheer R. McKenna J. T. Strecker R. E. McCarley R. W. (2012). Control of sleep and wakefulness. Physiol. Rev. 92, 1087–1187. 10.1152/physrev.00032.2011 22811426 PMC3621793

[B6] Chavez M. Besserve M. Adam C. Martinerie J. (2006). Towards a proper estimation of phase synchronization from time series. J. Neurosci. Methods 154, 149–160. 10.1016/j.jneumeth.2005.12.009 16445988

[B7] Chella F. Pizzella V. Zappasodi F. Marzetti L. (2016). Impact of the reference choice on scalp eeg connectivity estimation. J. Neural Eng. 13:036016. 10.1088/1741-2560/13/3/036016 27138114

[B8] Dang-Vu T. T. Schabus M. Desseilles M. Sterpenich V. Bonjean M. Maquet P. (2010). Functional neuroimaging insights into the physiology of human sleep. Sleep 33, 1589–1603. 10.1093/sleep/33.12.1589 21120121 PMC2982729

[B9] Dien J. (1998). Issues in the application of the average reference: review, critiques, and recommendations. Behav. Res. Methods Instrum. Comput. 30, 34–43. 10.3758/BF03209414

[B10] Dijk D. Brunner D. P. Borbely A. A. (1990). Time course of eeg power density during long sleep in humans. Am. J. Physiol. Regul. Integr. Compar. Physiol. 258, R650–R661. 10.1152/ajpregu.1990.258.3.R650 2316712

[B11] Dijk D.-J. Czeisler C. A. (1995). Contribution of the circadian pacemaker and the sleep homeostat to sleep propensity, sleep structure, electroencephalographic slow waves, and sleep spindle activity in humans. J. Neurosci. 15, 3526–3538. 10.1523/JNEUROSCI.15-05-03526.1995 7751928 PMC6578184

[B12] Fein G. Raz J. Brown F. F. Merrin E. L. (1988). Common reference coherence data are confounded by power and phase effects. Electroencephalogr. Clin. Neurophysiol. 69, 581–584. 10.1016/0013-4694(88)90171-X 2453336

[B13] French C. C. Beaumont J. G. (1984). A critical review of eeg coherence studies of hemisphere function. Int. J. Psychophysiol. 1, 241–254. 10.1016/0167-8760(84)90044-8 6394561

[B14] Gabor D. (1946). Theory of communication. part 1: the analysis of information. J. Inst. Electr. Eng. Part III 93, 429–441. 10.1049/ji-3-2.1946.0074

[B15] Geier C. Bialonski S. Elger C. E. Lehnertz K. (2015). How important is the seizure onset zone for seizure dynamics? Seizure 25, 160–166. 10.1016/j.seizure.2014.10.013 25468511

[B16] Goldman D. (1950). The clinical use of the average reference electrode in monopolar recording. Electroencephalogr. Clin. Neurophysiol. 2, 209–212. 10.1016/0013-4694(50)90039-3 15421286

[B17] Gordon R. Rzempoluck E. J. (2004). Introduction to laplacian montages. Am. J. Electroneurodiagnost. Technol. 44, 98–102. 10.1080/1086508X.2004.11079469 15328706

[B18] Guevara M. Lorenzo I. Arce C. Ramos J. Corsi-Cabrera M. (1995). Inter-and intrahemispheric EEG correlation during sleep and wakefulness. Sleep 18, 257–265. 10.1093/sleep/18.4.257 7618024

[B19] Hjorth B. (1975). An on-line transformation of eeg scalp potentials into orthogonal source derivations. Electroencephalogr. Clin. Neurophysiol. 39, 526–530. 10.1016/0013-4694(75)90056-5 52448

[B20] Hramov A. E. Koronovskii A. A. Kurovskaya M. K. Moskalenko O. I. (2005). Synchronization of spectral components and its regularities in chaotic dynamical systems. Phys. Rev. E 71:056204. 10.1103/PhysRevE.71.056204 16089631

[B21] Hu S. Lai Y. Valdes-Sosa P. A. Bringas-Vega M. L. Yao D. (2018). How do reference montage and electrodes setup affect the measured scalp eeg potentials? J. Neural Eng. 15:026013. 10.1088/1741-2552/aaa13f 29368697

[B22] Hu S. Stead M. Dai Q. Worrell G. A. (2010). On the recording reference contribution to EEG correlation, phase synchorony, and coherence. IEEE Trans. Syst. Man Cybern. B (Cybernetics) 40, 1294–1304. 10.1109/TSMCB.2009.2037237 20106746 PMC2891575

[B23] Jackson J. D. (1999). Classical Electrodynamics, 3rd Edn. New York, NY: John Wiley & Sons.

[B24] Jones B. E. (2019). Arousal and sleep circuits. Neuropsychopharmacology. 10.1038/s41386-019-0444-2. [Epub ahead of print]. 31216564 PMC6879642

[B25] Kantz H. Schreiber T. (2004). Nonlinear Time Series Analysis, Vol. 7. Cambridge, UK: Cambridge University Press.

[B26] Kayser J. Tenke C. E. (2010). In search of the rosetta stone for scalp EEG: converging on reference-free techniques. Clin. Neurophysiol. 121:1973. 10.1016/j.clinph.2010.04.030 20566375 PMC2953588

[B27] Kayser J. Tenke C. E. (2015). Hemifield-dependent n1 and event-related theta/delta oscillations: an unbiased comparison of surface laplacian and common eeg reference choices. Int. J. Psychophysiol. 97, 258–270. 10.1016/j.ijpsycho.2014.12.011 25562833 PMC4490127

[B28] Kreuz T. Mormann F. Andrzejak R. G. Kraskov A. Lehnertz K. Grassberger P. (2007). Measuring synchronization in coupled model systems: a comparison of different approaches. Physica D 225, 29–42. 10.1016/j.physd.2006.09.039

[B29] Laloux L. Cizeau P. Bouchaud J.-P. Potters M. (1999). Noise dressing of financial correlation matrices. Phys. Rev. Lett. 83:1467 10.1103/PhysRevLett.83.1467

[B30] Lei X. Liao K. (2017). Understanding the influences of eeg reference: a large-scale brain network perspective. Front. Neurosci. 11:205. 10.3389/fnins.2017.00205 28450827 PMC5390022

[B31] Lemos M. S. Fisch B. (1991). The weighted average reference montage. Electroencephalogr. Clin. Neurophysiol. 79, 361–370. 10.1016/0013-4694(91)90201-E 1718709

[B32] Lesser R. P. (1986). American EEG society guidelines in EEG and the evoked potentials. J. Clin. Neurophysiol. 3, 1–152. 3734059

[B33] Liu Q. Balsters J. H. Baechinger M. van der Groen O. Wenderoth N. Mantini D. (2015). Estimating a neutral reference for electroencephalographic recordings: the importance of using a high-density montage and a realistic head model. J. Neural Eng. 12:056012. 10.1088/1741-2560/12/5/056012 26305167 PMC4719184

[B34] Mormann F. Kreuz T. Rieke C. Andrzejak R. G. Kraskov A. David P.. (2005). On the predictability of epileptic seizures. Clin. Neurophysiol. 116, 569–587. 10.1016/j.clinph.2004.08.025 15721071

[B35] Mormann F. Lehnertz K. David P. Elger C. E. (2000). Mean phase coherence as a measure for phase synchronization and its application to the eeg of epilepsy patients. Physica D 144, 358–369. 10.1016/S0167-2789(00)00087-7

[B36] Müller M. Baier G. Galka A. Stephani U. Muhle H. (2005). Detection and characterization of changes of the correlation structure in multivariate time series. Phys. Rev. E 71:046116. 10.1103/PhysRevE.71.046116 15903735

[B37] Müller M. Jiménez Y. L. Rummel C. Baier G. Galka A. Stephani U.. (2006). Localized short-range correlations in the spectrum of the equal-time correlation matrix. Phys. Rev. E 74:041119. 10.1103/PhysRevE.74.041119 17155034

[B38] Müller M. F. Baier G. Jiménez Y. L. García A. O. M. Rummel C. Schindler K. (2011). Evolution of genuine cross-correlation strength of focal onset seizures. J. Clin. Neurophysiol. 28, 450–462. 10.1097/WNP.0b013e318231c894 21946370

[B39] Müller M. F. Rummel C. Goodfellow M. Schindler K. (2014). Standing waves as an explanation for generic stationary correlation patterns in noninvasive eeg of focal onset seizures. Brain Connect. 4, 131–144. 10.1089/brain.2013.0192 24494638

[B40] Nunez P. L. (2010). Rest: a good idea but not the gold standard. Clin. Neurophysiol. 121:2177 10.1016/j.clinph.2010.04.029 PMC2967671 20554245

[B41] Nunez P. L. Silberstein R. B. Shi Z. Carpenter M. R. Srinivasan R. Tucker D. M.. (1999). EEG coherency II: experimental comparisons of multiple measures. Clin. Neurophysiol. 110, 469–486. 10.1016/S1388-2457(98)00043-1 10363771

[B42] Nunez P. L. Srinivasan R. (2006). Electric fields of the Brain: The Neurophysics of EEG. New York, NY: Oxford University Press.

[B43] Nunez P. L. Srinivasan R. Westdorp A. F. Wijesinghe R. S. Tucker D. M. Silberstein R. B.. (1997). EEG coherency: I: statistics, reference electrode, volume conduction, laplacians, cortical imaging, and interpretation at multiple scales. Electroencephalogr. Clin. Neurophysiol. 103, 499–515. 10.1016/S0013-4694(97)00066-7 9402881

[B44] Offner F. (1950). The EEG as potential mapping. The value of the average monopolar reference. Electroencephalogr. Clin. Neurophysiol. 2, 213–214. 10.1016/0013-4694(50)90040-X 15421287

[B45] Olguín-Rodríguez P. V. Arzate-Mena J. D. Corsi-Cabrera M. Gast H. Marín-García A. Mathis J.. (2018). Characteristic fluctuations around stable attractor dynamics extracted from highly nonstationary electroencephalographic recordings. Brain Connect. 8, 457–474. 10.1089/brain.2018.0609 30198323

[B46] Osselton J. (1965). Acquisition of eeg data by bipolar unipolar and average reference methods: a theoretical comparison. Electroencephalogr. Clin. Neurophysiol. 19, 527–528. 10.1016/0013-4694(65)90195-1 4158666

[B47] Oswald J. (1956). The theory of analytic band-limited signals applied to carrier systems. IRE Trans. Circ. Theory 3, 244–251. 10.1109/TCT.1956.1086333

[B48] Pace-Schott E. F. Hobson J. A. (2002). The neurobiology of sleep: genetics, cellular physiology and subcortical networks. Nat. Rev. Neurosci. 3:591. 10.1038/nrn895 12154361

[B49] Picinbono B. (1997). On instantaneous amplitude and phase of signals. IEEE Trans. Sig. Process. 45, 552–560. 10.1109/78.558469

[B50] Plerou V. Gopikrishnan P. Rosenow B. Amaral L. A. N. Guhr T. Stanley H. E. (2002). Random matrix approach to cross correlations in financial data. Phys. Rev. E 65:066126. 10.1103/PhysRevE.65.066126 12188802

[B51] Plerou V. Gopikrishnan P. Rosenow B. Amaral L. A. N. Stanley H. E. (1999). Universal and nonuniversal properties of cross correlations in financial time series. Phys. Rev. Lett. 83:1471 10.1103/PhysRevLett.83.1471

[B52] Qin Y. Xu P. Yao D. (2010). A comparative study of different references for eeg default mode network: the use of the infinity reference. Clin. Neurophysiol. 121, 1981–1991. 10.1016/j.clinph.2010.03.056 20547470

[B53] Rappelsberger P. (1989). The reference problem and mapping of coherence: a simulation study. Brain Topogr. 2, 63–72. 10.1007/BF01128844 2641476

[B54] Rios Herrera W. A. Escalona J. Rivera López D. Müller M. F. (2016). On the estimation of phase synchronization, spurious synchronization and filtering. Chaos 26:123106. 10.1063/1.4970522 28039985

[B55] Rummel C. Baier G. Müller M. (2007). The influence of static correlations on multivariate correlation analysis of the EEG. J. Neurosci. Methods 166, 138–157. 10.1016/j.jneumeth.2007.06.023 17692927

[B56] Schindler K. Leung H. Elger C. E. Lehnertz K. (2006). Assessing seizure dynamics by analysing the correlation structure of multichannel intracranial EEG. Brain 130, 65–77. 10.1093/brain/awl304 17082199

[B57] Schindler K. A. Bialonski S. Horstmann M.-T. Elger C. E. Lehnertz K. (2008). Evolving functional network properties and synchronizability during human epileptic seizures. Chaos 18:033119. 10.1063/1.2966112 19045457

[B58] She S. Li H. Ning Y. Ren J. Wu Z. Huang R.. (2017). Revealing the dysfunction of schematic facial-expression processing in schizophrenia: a comparative study of different references. Front. Neurosci. 11:314. 10.3389/fnins.2017.00314 28620278 PMC5450627

[B59] Steriade M. (2003). The corticothalamic system in sleep. Front. Biosci. 8:d878–899. 10.2741/1043 12700074

[B60] Travis F. (1994). A second linked-reference issue: possible biasing of power and coherence spectra. Int. J. Neurosci. 75, 111–117. 10.3109/00207459408986294 8050845

[B61] Trujillo L. T. Stanfield C. T. Vela R. D. (2017). The effect of electroencephalogram (EEG) reference choice on information-theoretic measures of the complexity and integration of EEG signals. Front. Neurosci. 11:425. 10.3389/fnins.2017.00425 28790884 PMC5524886

[B62] Yao D. (2001). A method to standardize a reference of scalp EEG recordings to a point at infinity. Physiol. Meas. 22:693. 10.1088/0967-3334/22/4/305 11761077

[B63] Zaveri H. P. Duckrow R. B. Spencer S. S. (2000). The effect of a scalp reference signal on coherence measurements of intracranial electroencephalograms. Clin. Neurophysiol. 111, 1293–1299. 10.1016/S1388-2457(00)00321-7 10880805

[B64] Zaveri H. P. Duckrow R. B. Spencer S. S. (2006). On the use of bipolar montages for time-series analysis of intracranial electroencephalograms. Clin. Neurophysiol. 117, 2102–2108. 10.1016/j.clinph.2006.05.032 16887380

